# First Trimester Maternal Plasma Aberrant miRNA Expression Associated with Spontaneous Preterm Birth

**DOI:** 10.3390/ijms232314972

**Published:** 2022-11-29

**Authors:** Danai Mavreli, Mariana Theodora, Margaritis Avgeris, Nikolas Papantoniou, Panagiotis Antsaklis, George Daskalakis, Aggeliki Kolialexi

**Affiliations:** 1 Laboratory of Medical Genetics, School of Medicine, National and Kapodistrian University of Athens, 106 79 Athens, Greece; 2 1st Department of Obstetrics and Gynecology, School of Medicine, National and Kapodistrian University of Athens, 106 79 Athens, Greece; 3 Laboratory of Clinical Biochemistry–Molecular Diagnostics, Second Department of Pediatrics, School of Medicine, National and Kapodistrian University of Athens, “P. & A. Kyriakou” Children’s Hospital, 106 79 Athens, Greece; 4 Department of Genetics, Institute of Child Health, 106 79 Athens, Greece

**Keywords:** microRNAs, miRNAs, small RNA sequencing, small RNA-seq, sPTD, miR-23b-5p, miR-125a-5p, miR-4732-5p

## Abstract

Spontaneous Preterm Delivery (sPTD) is one of the leading causes of perinatal mortality and morbidity worldwide. The present case–control study aims to detect miRNAs differentially expressed in the first trimester maternal plasma with the view to identify predictive biomarkers for sPTD, between 32^0/7^ and 36^6/7^ weeks, that will allow for timely interventions for this serious pregnancy complication. Small RNA sequencing (small RNA-seq) of five samples from women with a subsequent sPTD and their matched controls revealed significant down-regulation of miR-23b-5p and miR-125a-3p in sPTD cases compared to controls, whereas miR-4732-5p was significantly overexpressed. Results were confirmed by qRT-PCR in an independent cohort of 29 sPTD cases and 29 controls. Statistical analysis demonstrated that miR-125a is a promising early predictor for sPTL (AUC: 0.895; 95% CI: 0.814-0.972; *p* < 0.001), independent of the confounding factors tested, providing a useful basis for the development of a novel non-invasive predictive test to assist clinicians in estimating patient-specific risk.

## 1. Introduction

Spontaneous Preterm Delivery (sPTD), defined as delivery that occurs before the completion of 37 weeks of gestation, is a complex pregnancy-related complication with multiple etiologies affecting 5–18% of all pregnancies worldwide [1]. 

sPTD is a leading cause of perinatal mortality and morbidity accounting for approximately 16% of all deaths and 35% of deaths among newborns. [2]. Premature infants that survive are at higher risk of developmental and neurological dysfunction later in life [3]. It is therefore widely accepted that the foresight in women at risk for sPTD early in pregnancy may enable, through earlier intervention, prolonged pregnancy and improved neonatal outcome [4]. 

Despite extensive efforts, screening strategies to accurately predict sPTD are still unsatisfactory. Currently, the most effective predictive tool combines well-known risk factors including maternal age, maternal BMI, racial origin, maternal behavior, spontaneous or assisted conception and prior history of sPTD, resulting in the identification of ~18% of nulliparous and 38% of parous sPTD cases, with a 10% false positive rate [5]. The prediction rate has been shown to increase by 54.8% by combining a priori risk factors with the measurement of cervical length [6]. 

MicroRNAs (miRNAs) are a class of non-coding RNAs of 19–25nt long responsible for regulating gene expression at the posttranscriptional level, without altering the genetic code. Concerning mode of action, miRNAs target the 3′ untranslated region (UTR) of mRNAs, resulting in translational inhibition and finally in mRNA degradation [7]. miRNAs have been documented to modulate most biological processes, such as cell proliferation, survival, differentiation and apoptosis. Interestingly, miRNAs can be detected within the cells, but they are also released in a stable form, due to their small size, into body fluids, such as plasma and serum [8]. 

Often, altered expression levels of circulating miRNAs have been involved with the initiation and progression of various diseases, including pregnancy complications, suggesting their role as potential biomarkers. 

Regarding sPTD, several studies have investigated circulating miRNAs as potential biomarkers for its prediction. However, most studies were restricted to miRNAs associated to cardiovascular disease, clusters from chromosome 14 (C14MC), chromosome 19 (C19MC) and miR-371-3 cluster or were performed using samples collected later during pregnancy, in the second or even the third trimester [9,10,11,12,13,14,15]. However, an ideal biomarker would be detectable during the first trimester of pregnancy, when routine screening for fetal aneuploidies is also offered, to reduce anxiety throughout pregnancy in low-risk women and allow for the close monitoring and timely interventions in the high-risk group [16].

The aim of the present study is to identify differentially expressed miRNAs in first trimester maternal plasma and to evaluate their clinical value as novel biomarkers for the early prediction of sPTD. Analysis was performed using small RNA next-generation sequencing (small RNA-seq) followed by quantitative real-time polymerase chain reaction (qRT-PCR) to confirm the results. The sPTD group consisted of women who experienced premature delivery between 32^0/7^ and 36^6/7^ weeks of gestation (moderate/late sPTD), which represent more than 8% of all premature deliveries [17]. Born at this gestational age, premature neonates, are at a relatively lower risk of mortality and morbidity than early preterm births. Still, the impact on healthcare worldwide is significant due to their increased risks compared to full-term births [18].

## 2. Results 

### 2.1. Demographic and Clinical Characteristics 

Table 1 describes the comparison between the demographic and the clinical characteristics of the cases and controls. Beside pre-pregnancy BMI, no significant differences were noted between the two groups.

### 2.2. Small RNA-seq Analysis 

Differentially expressed miRNAs were screened using small RNA-seq in five pairs of plasma samples. A total of 1682 unique circulating miRNAs were identified across all first trimester maternal plasma samples analyzed. Among these, 387 miRNAs were up-regulated and 95 miRNAs were down-regulated in sPTD cases compared to controls. Three circulating miRNAs showed significant changes (*p*-Value < 0.05, FC >1.5) in the sPTD group as compared to the control group. Specifically, significantly decreased levels of miR-23b-5p (*p* < 0.047, FC = 0.60) and miR-125a-3p (*p* = 0.014, FC = 0.59) were detected during the first trimester of gestation in women who subsequently experienced sPTD between 32^0/7^ and 36^6/7^ weeks of gestation whereas miR-4732-5p was significantly overexpressed *(p* = 0.048, FC = 1.70) (Supplement S1).

**Figure 1:**
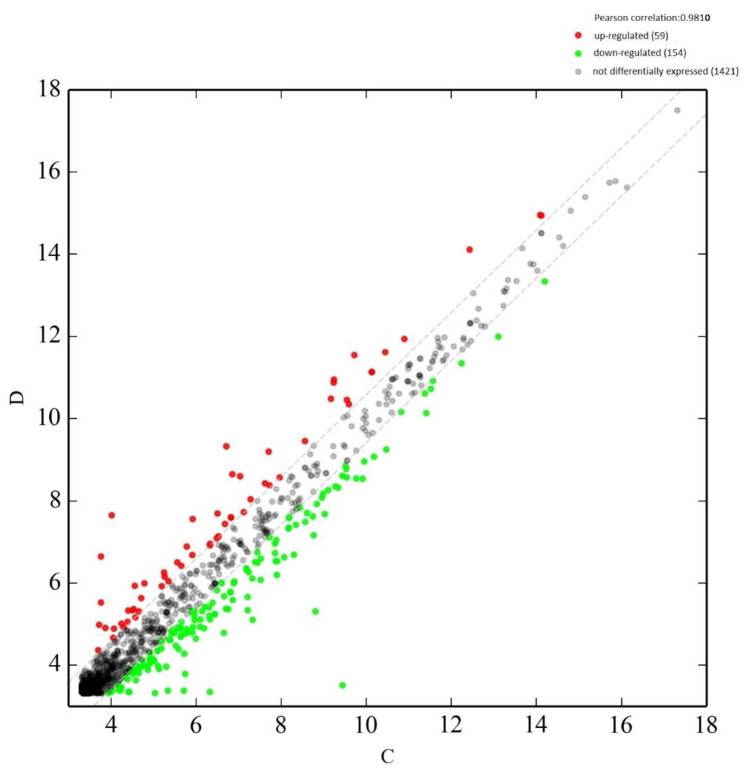
Scatter plot of circulating miRNA levels in the first trimester maternal plasma from women who subsequently experienced sPTD when compared to term ones. Red points show up-regulated miRNAs and green points show down-regulated miRNAs. Data from the sPTD group are plotted on the vertical axis (*y*-axis). Data from the term deliveries (controls) are plotted on the horizontal axis (*x*-axis).

**Figure 2:**
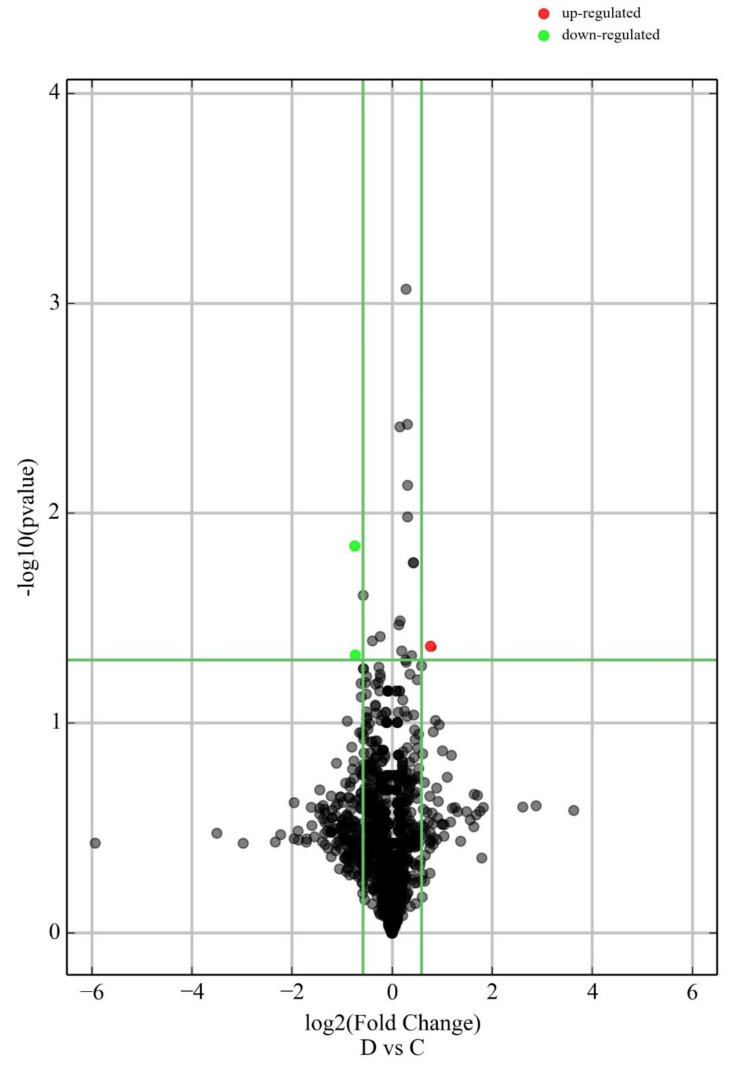
Volcano plot illustrates significantly differentially abundant circulating miRNAs. The -log10 (Benjamini–Hochberg corrected *p*-Value) is plotted against the log2 (fold change: sPTD cases/controls). The non-axial vertical lines denote ±1.5-fold change while the non-axial horizontal line denotes *p*-Value = 0.05, which is our significance threshold (prior to logarithmic transformation).

A scatter plot is used to display differentially expressed circulating RNAs (Figure 1). Significantly dysregulated circulating miRNAs are presented in a volcano plot (Figure 2).

### 2.3. GO Analysis 

The downstream targets of the significantly dysregulated miRNAs were identified commonly between Targetscan and miRDB databases and used for GO analysis. The significant biological processes associated with these miRNA target genes are presented in Table 2. KEGG pathway analysis revealed that the significantly underexpressed miRNAs target signaling pathways associated with the T cell receptor signaling pathway, Homologous recombination and Osteoclast differentiation (Table 3). No significant pathways were identified for the target genes of the up-regulated miRNAs.

### 2.4. qRT-PCR Assays

**Figure 3:**
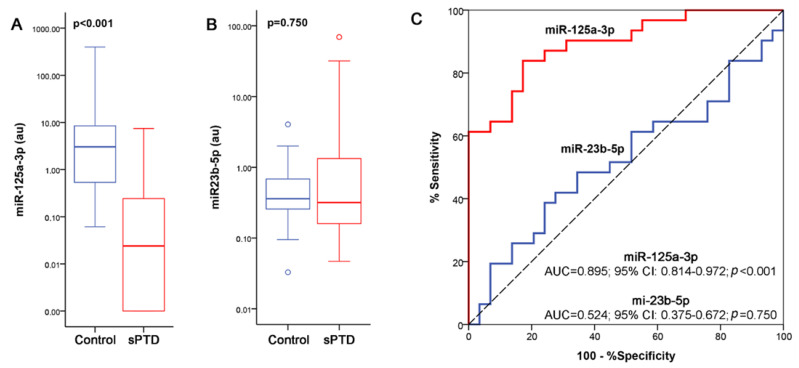
Statistical analysis of the data obtained using qRT-PCR. (**A**,**B**): Box-plots of the expression levels of miR-23b and miR-125a in the first trimester maternal plasma in women with sPTD versus uncomplicated pregnancies based on data derived from qRT-qPCR. *p*-Values calculated by Mann–Whitney U test. (**C**): ROC curve analysis for the prediction of sPTD based on miRNAs levels. The diagram is a plot of the % sensitivity (true-positive rate) vs. 100% specificity (false-positive rate).

The expression levels miR-23b-5p and miR-125a-3p were further determined in an independent cohort of 58 first trimester maternal plasma samples (29 sPTD cases and 29 controls) by qRT-PCR in order to confirm the reliability of the small RNA-seq analysis. The selection of miRNAs for verification by qRT-PCR was based on *p*-Values (Supplement S1). When compared with the control group, miR-125a-3p was significantly down-regulated in sPTD cases (*p* < 0.001; Figure 3A); where for miR-23b-5p, no statistically significant changes were observed (*p* = 0.750; Figure 3B). The qRT-PCR data were further used to assess the ability of miRNAs to discriminate between women at risk for sPTD and controls using ROC analysis. As expected, miR-125a-3p was highlighted to differentiate sPTD cases from uncomplicated pregnancies (AUC: 0.895; 95% CI: 0.814–0.972; *p* < 0.001; Figure 3C). Binomial logistic regression analysis demonstrated that miR-125a-3p represents a significant predictor of pregnancy outcome independent of the confounding factors tested (Table 4). 

## 3. Discussion

In the current study, we performed small RNA-seq in first trimester maternal plasma samples to investigate whether a differential miRNA expression profile is associated with subsequent sPTD and to provide a useful basis for the development of a novel non-invasive predictive test to assist clinicians in estimating patient-specific risks. 

Compared to women who delivered at term, maternal plasma miRNA profiling of women who subsequently developed sPTD revealed significant alterations in the expression level of miR-23b-5p, mIR-125a-5p and mIR-4732-5p. It is noteworthy that none has previously been related to sPTD. Downstream targets, however, have been associated with biological processes with an important role in the pathogenesis of sPTD, e.g., immune response, inflammation and apoptosis.

More precisely, miR-4732-5p showed a significant over expression in the first trimester maternal plasma of women destined to deliver sPTD compared to those with uncomplicated pregnancies. miR4732-5p has a proven role in tumorigenesis [19,20]. However, our GO analysis revealed that its downstream targets include members of suppressors of the cytokine signaling (SOCS) family, namely SOCS1-SOCS7. The SOCS protein family is implicated in the negative regulation of cytokine signaling and therefore in the regulation of pregnancy and labor [21,22]. Blumenstein et al. reported overexpression of SOCS1, SOCS2 and SOCS3 proteins in human placenta in an attempt to either prevent the entrance of pathogens into the maternal circulation or suppress trophoblast cytokine production and inhibit their damaging effects on the fetus [23]. In the control group, i.e., women who delivered at term, a decrease in SOCS1 and SOCS3 was observed possibly reflecting the involvement of inflammatory cytokines in mechanisms leading to delivery. However, no alteration in SOCS protein levels was demonstrated in the sPTD group. Still, further investigation is required to uncover the relationship between circulating miR-4732-5p and SOCS proteins as the lack of change in SOCS levels in the sPTD group could be attributed to immaturity. 

Moreover, 23b-5p, a pleiotropic miRNA demonstrating diverse effects on various biological processes, was significantly down-regulated in the first trimester maternal plasma in women who later delivered prematurely as compared to uncomplicated pregnancies. miR-23b is a member of the miR-23-27-24 family which consists of two paralogs with the miR-23a cluster (miR-23a-27a-24-2) found on chromosome 19 and the intragenic miR-23b cluster (miR-23b27b-24-1) located on chromosome 9 within the C9orf3 gene [24]. Previous research suggested that miR-23b plays vital roles in cancer development where it exerts either oncogenic or tumor suppressor activity [25,26]. It is also implicated in the regulation of angiogenesis and endothelial cells homeostasis and may serve as biomarkers for the early diagnosis of acute myocardial infarction [27,28]. Moreover, research findings suggest that miR-23b overexpression enhanced the expression of IL-10 which is vital for normal pregnancy, and low IL-10 levels are associated with pregnancy complications [29]. Hence, down-regulation of miR-23b has been reported in first trimester maternal plasma in women who later developed late onset pre-eclampsia as compared to uncomplicated pregnancies, indicating a possible involvement in the pathogenesis of the condition [24].

Finally, miR-125a-5p is involved in development and cell differentiation and therefore it is implicated in several malignancies including gastric, cervical, lung and ovarian cancers, retinoblastoma and neuroblastoma [30,31,32]. Recently, it was demonstrated that miR-125a suppresses cell proliferation and migration and inhibits angiogenesis by regulating its downstream target vascular endothelial growth factor A (VEGFA), indicating a potential role to the pathophysiology of pre-eclampsia [33].

Down-regulation of miR-125a was detected in the discovery cohort using small RNA-seq and was further confirmed by the verification cohort. Following statistical analysis, the AUC for miR-125a-5p was 89%, demonstrating promising diagnostic potential as a first trimester screening test for the prediction of subsequent sPTD at 33–36 weeks of gestation. Furthermore, logistic regression analysis revealed that miR-125a-5p is an early predictor of sPTD independently of well-known risk factors (previous sPTD, maternal smoking, maternal pre-pregnancy BMI, maternal age, fetal gender and mode of conception).

Hence, once validated in a large cohort, miR-125a-5p might be used to identify women at high risk for sPTD, allowing for close monitoring and/or clinical interventions such as cervical cerclage. They can also assist the development of novel therapeutics. To date, although the role of miRNA therapeutics has not yet been translated into clinical practice, miRNA mimics and miRNA suppressors have shown significant efficiency in various cancers [34]. 

To the best of our knowledge, this is the first study that aimed to identify novel biomarkers for the early prediction of women at risk for sPTD using a sensitive high-throughput method for miRNA plasma profiling followed by verification of the results obtained using RT-qPCR. We acknowledge, however, that the small sample size poses a significant limitation for our study. 

Still, this pilot study aims to offer improved candidate biomarkers predictive of a subsequent sPTD to be validated in a larger series of pregnant women. 

## 4. Materials and Methods

### 4.1. Study Population

Maternal samples for this retrospective study for sPTD were collected from pregnant women during first trimester prenatal screening for fetal aneuploidies between March 2018 and December 2020 as previously described [35]. At the same time, maternal demographic characteristics and medical history were obtained and recorded in an electronic database. Gestational age was determined based on the last menstrual period and confirmed by ultrasound measurement of the fetal crown rump length (CRL). Pregnancy outcomes were made known after the completion of pregnancies from the maternity hospital files and were also recorded in the database. 

The selection of samples for analysis was carried out using a case–control design. Cases were pregnant women with a subsequent sPTD, between 32^0/7^ and 36^6/7^ weeks of gestation. The control group consisted of participants with termly delivered neonates. Cases and controls were matched with respect to maternal age and duration of storage at −80°. Only European women with singleton pregnancies who delivered a phenotypically normal live born neonate were included in the study. Women with missing information or insufficient plasma for analysis, those with chronic diseases or pregnancy complications other than sPTD (e.g., pre-eclampsia, gestational diabetes, fetal growth restriction) as well as women with PPROM or signs of intra-uterine infection/inflammation at the time of admission were excluded from the study.

Over the study period, a total of 1809 plasma samples were collected and stored at −80 °C. Through the database search, 63 women were identified to be diagnosed with sPTD. Of those, 28 were excluded from the study because of fetal chromosomal abnormality/major fetal malformations (n = 4), presence of a multiple pregnancy (n = 6), miscarriage or fetal death before 24 weeks or termination (n = 5), lost to follow-up (n = 4) or due to inadequate plasma sample (n = 9). Plasma sample of a pregnant woman who subsequently delivered preterm before the 32nd week of gestation (early sPTD) was excluded to ensure increased homogeneity among cohort. Different aliquots from the same cohort have been used previously in a proteomic study [35].

Finally, 34 plasma samples from women who subsequently delivered prematurely, between 32^0/7^ and 36^6/7^ weeks, fulfilled the inclusion criteria and were retrieved for analyses along with their matched controls. Selected samples were analyzed in two phases: samples from five women with a subsequent sPTD between 32^0/7^ and 36^6/7^ weeks of gestation and with their matched controls were analyzed using small RNA-seq (discovery cohort) to identify miRNAs with significantly aberrant expression levels between the compared groups. Based on discovery cohort data, the remaining 58 samples (29 sPTL cases and 29 controls) were used in the second phase of the study to confirm the differential expression of selected miRNAs. None of the samples were previously thawed and refrozen.

Written informed consent to collect and use the biological samples and clinical information for research purposes was obtained from all participants prior to inclusion in the study. The study was approved by Alexandra’s Hospital ethics committee (P.N. 9/5-1-2018) and conducted according to the standards of the 1975 Declaration of Helsinki, as revised in 2008.

### 4.2. Clinical Definitions

sPTD is defined by the presence of regular uterine contractions (at least two uterine contractions every 10 min for 30 min, as confirmed by external tocometry) in combination with cervical changes occurring prior to 36^6/7^ weeks that required hospitalization [36]. A term pregnancy is defined as a delivery from 37 completed weeks to less than 42 and was used to describe the optimal timing for a good outcome for the mother and baby [37]. 

### 4.3. Methods

#### 4.3.1. Total RNA and miRN Isolation

Total RNA enriched in miRNAs was extracted from EDTA-preserved maternal plasma using the mirVana miRNA Isolation kit (Thermo Fisher, Waltham, MA, USA) according to the manufacturer’s instructions. The RNA integrity was determined with polyacrylamide gel electrophoresis. The concentration and purity of RNA were checked by NanoDrop ND-1000 spectrophotometer (Thermo Fisher, Wilmington, DE, USA) and 2100 Bioanalyzer Instruments (Agilent, Santa Clara, CA, USA).

#### 4.3.2. Small RNA-seq

Profiling of circulating miRNA was performed by small RNA-seq in Illumina NextSeq 500 platform as previously described [24]. Briefly, small RNA libraries were generated using the TruSeq^®^ miRNA Sample Prep kit v2 (Illumina, San Diego, CA, USA). RNA template of each sample was sequentially ligated to 3′ and 5′ adapters, reverse transcribed, PCR amplified and selected on agarose gels by size of ~130–150 bp (correspond to ~15–35nt small RNAs). Then, PCR amplified fragments were eluted from gel pieces, purified and quantified by Agilent 2100 Bioanalyzer (Agilent, Santa Clara, CA, USA). The completed libraries were diluted to a final concentration of 8 pM and processed for cluster generation on the Illumina cBOT using TruSeq Rapid SR cluster kit (Illumina, San Diego, CA, USA). Sequencing was performed on Illumina NextSeq 500 platform using TruSeq Rapid SRB kits (Illumina, San Diego, CA, USA). 

#### 4.3.3. Sequencing Data Analysis

The complete raw sequences from Illumina NextSeq 500 were generated as clean reads by real-time base calling and quality filtering. The clean reads were recorded in FASTQ format, containing the read information, sequences and quality encoding. The 3′ prime adapter sequences were trimmed and reads with lengths <15 nucleotides were removed. Those with length >15nt were aligned to all human miRNAs (miRBase v21) using NovoAlign software (NovoCraft, Selangor, Malaysia) allowing maximum one mismatch per sequence [38]. The miRNA read counts were used to estimate the expression level of each miRNA. All counts were normalized by reads per million [39]. Differential expression and statistical analysis of sequencing data were performed by the DESeq2 package in R. 

Circulating miRNAs having *p*-Value ≤ 0.05 and FC ≥ 1.5 were considered significant. *p*-Values were adjusted for multiple hypotheses testing (<0.01 compared to control) using the method of Benjamini–Hochberg to establish a false discovery rate (FDR).

#### 4.3.4. miRNAs Target Gene Prediction and Gene Ontology Analysis 

Potential targets of the significantly dysregulated miRNAs were obtained using Targetscan7 (http://www.targetscan.org/vert_71/) and MirdbV5 (http://mirdb.org/miRDB/) algorithms accessed 21 January 2020. Only overlapping results between these databases were accepted as potential targets. 

Targets gene were submitted to the WebGestalt web-tool http://bioinfo.vanderbilt.edu/webgestalt/ accessed on 21 January 2020 for Gene Ontology (GO) annotation and Kyoto Encyclopedia of Genes and Genomes (KEGG) signaling enrichment analyses [40,41]. GO terms and KEGG pathways having an adjusted *p*-Value ≤ 0.05 were considered significant.

#### 4.3.5. Quantitative Real-Time Polymerase Chain Reaction Verification 

The expression levels of representative miRNAs, differentially expressed in the first trimester maternal plasma of women who later experienced sPTD as compared to the control group were verified in an independent cohort consisting of 58 plasma samples (n = 29 sPTL and n = 29 controls) using qRT-PCR. cDNA synthesis and qRT-PCR were performed using a TaqMan miRNA Reverse Transcription kit (Applied Biosystems, Inc., Foster City, CA 94404, USA) and individual TaqMan MiRNA assays (Applied Biosystems, Inc., USA), following the manufacturer’s recommendations. All reactions were run in triplicate in a LC480 LightCycler system (Roche GmbH, Rotkreuz, Switzerland). The miRNA gene expression was determined using the 2^−ΔΔCt^ method [42]. RNU44 (Applied Biosystems, Inc., Foster City, CA 94404, USA) was used for normalization purposes. 

#### 4.3.6. Statistical Analysis

Statistical analyses were conducted in IBM SPSS Statistics 20 software (IBM Corp., Armonk, NY, USA). Comparisons of maternal demographic and clinical characteristics between the two groups were compared using Pearson chi-square test for the evaluation of categorical variables or the Mann–Whitney *U* test for continuous variables. A two-tailed Fisher’s exact test was applied to test GO and pathway enrichment of the target genes of the significant differentially expressed miRNAs. Receiver Operating Characteristic curves (ROC) were applied to evaluate the diagnostic value of each miRNA using the qRT-qPCR data. Binomial logistic regression analyses were performed using the occurrence of sPTD as the dependent variable and miRNA expression levels, previous sPTD, maternal smoking, maternal pre-pregnancy BMI, maternal age, fetal gender and mode of conception as independent variables. *p*-Values < 0.05 were considered significant.

## 5. Conclusions

The present study demonstrated that small RNA profiling allows for the identification of novel biomarkers in the first trimester maternal plasma that can potentially be used in clinical practice for early, minimally invasive, prediction of sPTD. Analysis revealed for the first time that the expression levels of circulating, miR-125a-5p, miR-23b and miR-4732-5p are significantly different in plasma samples collected from women who subsequently experience sPTD, as compared to uncomplicated pregnancies, implying their critical roles in the pathology of sPTD. Further studies should be performed for a more complete understanding of the topic. More importantly, the results obtained in our cohort demonstrated that miR-125a-5p may be used as reliable independent biomarkers to predict poor pregnancy outcomes.

## Figures and Tables

**Table 1:** Maternal and neonatal characteristics of moderate/late sPTD cases and controls included in the study.

Characteristic	Controls (n = 34)	sPTD Cases (n = 34)	*p*-Value
Maternal age (y) Median (min–max)	31.00 (24.80–38.80)	30.65 (24.80–38.20)	0.898
Pre-pregnancy BMI (kg/m^2^) Median (min–max)	25.95 (22.00–31.20)	24.65 (20.90–31.90)	0.028
Cigarette smoker Yes No	12 22	8 26	0.40
Mode of conception Spontaneous In vitro fertilization	28 (82.4%) 6 (17.6%)	26 (76.4%) 8 (23.6%)	0.750
Parity Nulliparous Parous	17 (50%) 17 (50%)	14 (41.2%) 20 (58.8%)	0.616
Previous PTD No Yes	32 (94.1%) 2 (5.88%)	27 (79.4%) 7 (20.58%)	0.104
Mode of Delivery Vaginal Caesarean section	29 (85.2%) 5 (14.7%)	22 (64.7%) 12 (35.2%)	0.075
Neonatal Gender Male Female	18 (52.95%) 16 (47.05%)	16 (47.05%) 18 (52.95%)	0.95

**Table 2:** GO annotations of miRNA’s target genes.

Down-Regulated miRNAs			
GO.ID	Term	*p*-Value	Genes
GO:0048585	Negative regulation of response to stimulus	0.000	RGS4//IL10//NDFIP1//GPRC5A//STAP1//MTMR4//TMEM127//BRCA1//GREM2//HGS//STK38//FAP//TPT1
GO:0014854	Response to inactivity	0.001	MTMR4//IL10
GO:1900119	Positive regulation of execution phase of apoptosis	0.001	FAP//BOK
GO:0009968	Negative regulation of signal transduction	0.001	RGS4//GPRC5A//IL10//MTMR4//TMEM127//BRCA1//GREM2//HGS//STK38//STAP1//TPT1
GO:0043032	Positive regulation of macrophage activation	0.001	STAP1//IL10
GO:0010324	Membrane invagination	0.001	ABCA1//STAP1//HGS
GO:1900120	Regulation of receptor binding	0.002	GREM2//IL10
GO:0010648	Negative regulation of cell communication	0.002	RGS4//GPRC5A//IL10//MTMR4//TMEM127//BRCA1//GREM2//HGS//STK38//STAP1//TPT1
GO:0023057	Negative regulation of signaling	0.002	RGS4//GPRC5A//IL10//MTMR4//TMEM127//BRCA1//GREM2//HGS//STK38//STAP1//TPT1
GO:0070230	Positive regulation of lymphocyte apoptotic process	0.002	IL10//PDCD1
**Up-Regulated miRNAs**			
**GO.ID**	**Term**	***p*-Value**	**GENES**
GO:0006397	mRNA processing	0.008	RAVER2//LUC7L3//ALKBH5
GO:0045625	Regulation of T-helper 1 cell differentiation	0.009	SOCS5
GO:0071071	Regulation of phospholipid biosynthetic process	0.009	HTR2A
GO:0002829	Negative regulation of type 2 immune response	0.010	SOCS5
GO:0006054	N-acetylneuraminate metabolic process	0.010	CMAS
GO:0007175	Negative regulation of epidermal growth factor-activated receptor activity	0.010	SOCS5
GO:0035970	Peptidyl-threonine dephosphorylation	0.010	PPM1E
GO:0042118	Endothelial cell activation	0.010	SOCS5
GO:0045623	Negative regulation of T-helper cell differentiation	0.010	SOCS5
GO:0043371	Negative regulation of CD4-positive, alpha-beta T cell differentiation	0.011	SOCS5

**Table 3:** Pathway enrichment analysis of the target genes of down-regulated miRNAs.

Pathway ID	Definition	*p*-Value	FDR	Enricment Score	Gene Ratio	Genes
hsa04660	T cell receptor signaling	0.0021	0.658	2.671681	0.16	IL10//PDCD1//TEC
hsa03440	Homologous recombination	0.0046	0.713	2.335566	0.11	BRCA1//FAM175A
hsa04380	Osteoclast differentiation	0.0425	1	1.37106	0.11	SIRPA//TEC

**Table 4:** Logistic regression analysis for the prediction of sPTD patients according to maternal. plasma miRNAs expression levels.

	Univariate Analysis		
Covariant	OR ^a^	95% CI ^b^	*p*-Value ^c^
miR-23b-5p	1.057	0.943–1.184	0.343
miR-125a-3p	0.654	0.490–0.872	0.004
Previous PTD	7.154	0.809–63.30	0.077
Smoking	0.535	0.176–1.624	0.269
Maternal pre-pregnancy BMI	0.808	0.650–1.003	0.053
Mode of conception Natural IVF	1.00 1.512	0.425–5.384	0.523
Fetal gender Male Female	1.00 1.319	0.470–3.703	0.600
Maternal age	1.016	0.885–1.167	0.819
	Multivariate analysis ^d^		
Covariant	OR ^a^	95% CI ^b^	*p*-Value ^c^
miR125a-3p	0.491	0.295–0.817	0.006

^a^ Odds Ratio. ^b^ 95% Confidence Interval of the estimated OR. ^c^ calculated by test for trend. ^d^ Multivariate models adjusted for tested miRNAs, previous PTD, smoking, BMI, mode of conception, fetal gender and age.

## Data Availability

The datasets used and/or analyzed during the current study are available from the corresponding author on reasonable request. Please also refer to the Section 4 “Materials and Methods”.

## Supplementary Materials

The following supporting information can be downloaded at: https://www.mdpi.com/article/10.3390/ijms232314972/s1. Supplement S1. MiRNAs with significant altered expression in the 1st trimester maternal plasma of women who later delivered preterm compared to term deliveries.
