# Supplementary material for: First Trimester Maternal Plasma Aberrant miRNA Expression Associated with Spontaneous Preterm Birth
Source: Int J Mol Sci. 2022 Nov 29;23(23):14972. doi: 10.3390/ijms232314972 (PMC9735892; doi:10.3390/ijms232314972)
Supplement: Supplementary file 1 [file ijms-23-14972-s001.zip › ijms-2035128-supplementary.pdf]

Supplement S1. MiRNAs with significant altered expression in the 1st trimester maternal plasma of women who later delivered preterm compared to term deliveries

		Tag Count		sPTD vs Controls		
**MATURE-ID**	**MATURE-SEQ**	**sPT**	**Controls**	**Fold change**	**p value**	**FDR**
Up-regulated						
hsa-miR-4732-5p	UGUAGAGCAGGGAGCAGGAAGCU	102.6	60.	1.70	0.04812	0.666
Down regulated						
hsa-miR-23b-5p	UGGGUUCCUGGCAUGCUGAUUU	14.2	23.6	0.60	0.04751	0.666
hsa-miR-125a-3p	ACAGGUGAGGUUCUUGGGAGCC	24.6	41.2	0.59	0.01435	0.666
